# Seroprevalence of *Bordetella pertussis* antibodies in adults in Hungary: results of an epidemiological cross-sectional study

**DOI:** 10.1186/s12879-017-2356-2

**Published:** 2017-04-04

**Authors:** Péter Torzsa, Raghavendra Devadiga, Monica Tafalla

**Affiliations:** 1 Faculty of Medicine, Department of Family Medicine, Semmelweis University, Kútvölgyi út 4, Budapest, 1125 Hungary; 2 GSK, CDOC-B, #5 Embassy Links, S.R.T. Road, Bangalore, 560052 India; 3 GSK, Avenue Fleming 20, Wavre, 1300 Belgium

**Keywords:** Seroprevalence, *Bordetella pertussis*, Epidemiology, Hungary

## Abstract

**Background:**

Pertussis (whooping cough) is well known to be underreported, particularly among adults, who can act as an infectious reservoir, potentially putting susceptible newborns at risk of serious illness. The purpose of this study was to estimate the seroprevalence of pertussis in adults in Hungary.

**Methods:**

This epidemiological, cross-sectional study was conducted in adults in five general practitioners’ practices in Hungary. Serum anti-pertussis toxin immunoglobulin G (anti-PT IgG) antibody levels were analyzed using enzyme-linked immunosorbent assay. Sera were classified following manufacturer’s instructions as: strongly indicative of current/recent infection (≥1.5 optical density [OD] units); indicative of current/recent infection (≥1.0 OD units); seropositive (>0.3 OD units); or seronegative (≤0.3 OD units). Logistic regression was performed to describe the associations between seroprevalence and various characteristics.

**Results:**

Between 24^th^ April 2014 and 24^th^ April 2015, 1999 adults (60.6% female; mean age 47.4 ± 17.7 years) were included in the analysis. A total of 14.8% were seropositive for anti-PT IgG, 1.1% had a level indicative of current/recent infection, and 0.1% had a level strongly indicative of current/recent infection. Logistic regression showed significant relationships between increased rates of seropositivity and: age ≥60 years (odds ratio [OR], 1.97; 95% confidence interval [CI], 1.39–2.80; *p* = .0002) or 18–29 years (OR, 1.67; 95% CI, 1.13–2.46; *p* = .0094) vs. 45–59 years; former smoker (OR, 1.46; 95% CI, 1.08–1.97; *p* = .014) or current smoker (OR, 1.38; 95% CI, 1.01–1.89; *p* = .045) vs. never smoker; and male (OR, 1.30; 95% CI, 1.01–1.68; *p* = .041) vs. female. Also, between increased rates of probable current/recent infection and current smoker (OR, 7.50; 95% CI, 2.32–24.31; *p* = .0008) or former smoker (OR, 4.07; 95% CI, 1.21–13.64; *p* = .023) vs. never smoker.

**Conclusions:**

Approximately 85% of the adults studied were seronegative and therefore susceptible to pertussis infection. Approximately 1% had anti-PT IgG levels indicative of current/recent pertussis infection, which could potentially be transmitted to susceptible young infants. Vaccination of adults is a key way to indirectly protect infants.

**Trial registration:**

Clinical Trials.gov NCT02014519. Prospectively registered 12 December 2013.

## Background

Pertussis (whooping cough) is an acute respiratory tract infection caused by *Bordetella pertussis* that is characterized by a chronic, severe cough [1]. As pertussis is a potentially life-threatening infection for newborns and unvaccinated infants [2], vaccination during infancy is recommended. In Hungary, pertussis immunization – using a combined diphtheria-tetanus-whole cell pertussis vaccine – was introduced in 1953 [3]. This had a huge beneficial impact on pertussis incidence [3]. In 2006, this was switched to a combined diphtheria-tetanus-acellular pertussis vaccine [4]. The optimum immunization schedule for pertussis is unclear, so these vary by country; in Hungary, the current recommended immunization schedule includes acellular pertussis at ages 2, 3, and 4 months, with boosters at ages 1.5, 6, and 11 years [5]. Coverage of all doses in Hungary is very high (>99%) [6].

However, adults are at risk of pertussis infection due to waning immunity following immunization or natural infection [7–10]. Although pertussis during adulthood is generally not serious, it can result in morbidity and – more importantly – infected adults can pass on the infection to infants who have not been (fully) vaccinated [9]. Some European countries have therefore introduced various combinations of: adolescent, adult, or elderly boosters; a “cocooning strategy” (vaccination of household members in contact with a newborn); or vaccination of pregnant women (to provide the infant with placentally transmitted antibodies) [11, 12]. Apart from the introduction of adolescent boosters, these strategies have not yet been recommended in Hungary [11]. Given that the notification rate in infants (age <1 year) in Hungary was 2.24/100,000 in 2014, roughly 12-fold higher than in those aged ≥1 year (approximately 0.18/100,000) [13], young infants are clearly still at risk of pertussis despite high pediatric population vaccination coverage.

Although overall pertussis notification rates in Hungary are among the lowest in Europe [13], at 0.17/100,000 in 2015 [14, 15], it is well known that pertussis is underreported, particularly among adults, who generally have much milder symptoms than infants, making it difficult to distinguish from a common cold [16]. The low notification rate of pertussis coupled with the high immunization coverage may result in general practitioners (GPs) in Hungary not testing adults with chronic cough for pertussis. The true incidence of pertussis may therefore be considerably higher than the notified cases, particularly among adults.

A more realistic estimate of pertussis incidence can be obtained from seroepidemiological studies; which can also help in the determination of immunity duration, the need for booster doses, and to investigate disease resurgence and its causes [17]. However, although high antibody titers are indicative of recent infection, lower levels can be due to more distant infection or vaccination. Although higher anti-pertussis toxin immunoglobulin G (anti-PT IgG) levels have been correlated with protection against pertussis [18], there is no agreement on the level of pertussis antibodies that confers protection against pertussis. This makes the interpretation of antibody prevalence data challenging. A number of studies have examined the seroprevalence of pertussis antigens in various countries, but as no such studies have yet been undertaken in Hungary, the purpose of this seroepidemiological study was to estimate the seroprevalence of pertussis in adults in Hungary.

## Methods

This epidemiological, cross-sectional study (NCT02014519) was conducted in adults in five centers in Hungary. Subjects were recruited from GP clinics between 24^th^ April 2014 and 24^th^ April 2015, mostly during a clinic visit for another purpose, but some by telephone or email contact (particularly in the youngest age group, due to difficulties in recruitment). Subjects had to be aged ≥18 years and provide written informed consent; and not have been vaccinated against pertussis within the previous 12 months or have a confirmed or suspected immunological disorder.

Serum anti-PT IgG levels were determined using a commercial enzyme-linked immunosorbent assay (ELISA) (*PERTUSSCAN PT IgG*, Euro Diagnostica AB, Sweden) at the Laboratory of the National Epidemiology Centre using standardized and validated procedures in accordance with the manufacturer’s instructions [19]. Test results were used to classify sera according to manufacturer pre-defined cut-off values as: strongly indicative of current/recent infection (≥1.5 optical density [OD] units; corresponds to 110 ELISA units [EU]/mL against the United States (US) Food and Drug Administration standard Lot 3 [19]); indicative of current/recent infection (≥1.0 OD units [70 EU/mL]); seropositive (>0.3 OD units [18 EU/mL]; the sensitivity limit of the assay); or seronegative (≤0.3 OD units).

The main aim of the study was to assess the seroprevalence of anti-PT IgG in adults in Hungary, according to the cut-off levels defined above; this was also assessed according to age category (18–29, 30–44, 45–59, and ≥60 years). Comparisons of seropositivity and anti-PT IgG levels indicative of current/recent infection by age category were tested using the Chi-square test. Relationships between seropositivity and age category, gender, history of vaccination against pertussis, history of pertussis infection, medication (antibiotics related to respiratory infections, prescribed and over-the-counter cough medicines) in the previous 12 months, hospitalization due to respiratory infections in the previous 12 months, and smoking status were examined by a logistic regression analysis (using the method of maximum likelihood to estimate the parameters). The same was done for antibody levels indicative of current/recent infection. In addition to the saturated models (i.e., considering all of the potential factors), final models using the backward elimination strategy (in which a *p*-value of 0.20 is required to keep a potential factor in the model) were also performed. Both models adjusted for all other factors in the model, including confounders. A *p*-value <.05 was taken to be statistically significant. All statistical analyses were performed using *SAS* Version 9.2.

The target sample size was approximately 2000 subjects (400 aged 18–29 years, 550 aged 30–44 years, 510 aged 45–59 years, 540 aged ≥60 years), based on a previous similar study in Israel [20].

## Results

A total of 1999 subjects valid for analysis were enrolled (one subject was excluded for violation of inclusion and exclusion criteria). Demographics are shown in Table 1. It should be noted that 836 subjects did not know their vaccination status. Of those who knew whether they had received a pertussis vaccination, 75.8% had been vaccinated. Only 5.6% of subjects who knew their pertussis history reported having been previously diagnosed with pertussis, most often as a child (86.2%). Among those who were seropositive, none reported having been previously diagnosed with pertussis as an adult. Among those who reported a long-lasting cough in the last 12 months (8.3%), most (86.4%) reported that their cough had lasted for ≥30 days and were dry (54.3%). Contact with a person with a long-lasting cough in the previous 12 months was reported by 12.6% of subjects, mainly with a family member (61.7%) and daily contact (67.8%). Only 0.5% of the subjects had been hospitalized due to respiratory infections in the previous 12 months, a median (range) of 1 (1–5) times, for a median (range) of 9 (3–35) days.

**Table 1:** Characteristics of the study population, overall and by seropositivity for anti-PT IgG

	Total (*n* = 1999)	Seropositive^a^ (*n* = 295)	Seronegative^b^ (*n* = 1704)
Demographics			
Age (years)	47.4 ± 17.7	48.8 ± 19.4	47.1 ± 17.4
Female	1212/1999 (60.6)	160/295 (54.2)	1052/1704 (61.7)
Pertussis-related history			
Pertussis vaccination	882/1163 (75.8)	127/165 (77.0)	755/998 (75.7)
Prior pertussis	88/1566 (5.6)	15/227 (6.6)	73/1339 (5.5)
Infant	3/87 (3.4)	0	3/73 (4.1)
Child	75/87 (86.2)	13/14 (92.9)	62/73 (84.9)
Adolescent	3/87 (3.4)	1/14 (7.1)	2/73 (2.7)
Adult	6/87 (6.9)	0	6/73 (8.2)
Long-lasting cough^c^	164/1971 (8.3)	20/290 (6.9)	144/1681 (8.6)
Recent contact with a person with pertussis/long-lasting cough^c^	227/1795 (12.6)	30/270 (11.1)	197/1525 (12.9)
Regular contact with children	919/1999 (46.0)	122/295 (41.4)	797/1704 (46.8)
Relevant medications^d^	241/1962 (12.3)	39/287 (13.6)	202/1675 (12.1)
Relevant hospitalizations^e^	10/1994 (0.5)	4/294 (1.4)	6/1700 (0.4)

Data are mean ± SD or *n*/*N* (%), where *N* is the number of subjects with known data

*Abbreviations: Anti-PT IgG* anti-pertussis toxin immunoglobulin G, *OD* optical density, *SD* standard deviation

^a^>0.3 OD units

^b^≤0.3 OD units

^c^Lasting ≥3 weeks in the previous 12 months

^d^Any antibiotics and/or other medication (i.e., any cough medicines) for lower respiratory tract infections or (suspected) pertussis infections in the previous 12 months

^e^Hospitalized due to respiratory infections in the previous 12 months

A total of 295 subjects (14.8%) were seropositive for anti-PT IgG (>0.3 OD units), 22 (1.1%) had a level indicative of current/recent infection (≥1.0 OD units), and 2 (0.1%) had a level strongly indicative of current/recent infection (≥1.5 OD units) (Table 2). Seropositivity (>0.3 OD units) varied significantly across age groups (*p* = .0004), and was the highest among those aged ≥60 years and 18–29 years; but current/recent pertussis infection status (≥1.0 OD) did not vary significantly by age (*p* = .096).

**Table 2:** Subjects with anti-PT IgG levels above/below the various cut-offs

Anti-PT IgG level	Total (*n* = 1999)	Age groups			
		18–29 years (*n* = 408)	30–44 years (*n* = 541)	45–59 years (*n* = 509)	≥60 years (*n* = 541)
Strongly indicative of current/recent infection (≥1.5 OD units)	2 (0.10 [0.01–0.36])	0 (0.00 [0.00–0.90])	1 (0.18 [0.00–1.03])	1 (0.20 [0.00–1.09])	0 (0.00 [0.00–0.68])
Indicative of current/recent infection (≥1.0 OD units)	22 (1.10 [0.69–1.66])	2 (0.49 [0.06–1.76])	5 (0.92 [0.30–2.14])	4 (0.79 [0.21–2.00])	11 (2.03 [1.02–3.61])
Seropositive (>0.3 OD units)	295 (14.76 [13.23–16.39])	67 (16.42 [12.96–20.38])	67 (12.38 [9.73–15.46])	56 (11.00 [8.42–14.05])	105 (19.41 [16.16–23.00])
Seronegative (≤0.3 OD units)	1704 (85.24 [83.61–86.77])	341 (83.58 [79.62–87.04])	474 (87.62 [84.54–90.27])	453 (89.00 [85.95–91.58])	436 (80.59 [77.00–83.84])

Data are *n* (% [95% CI])

*Abbreviations: Anti-PT IgG* anti-pertussis toxin immunoglobulin G, *CI* confidence interval, *OD* optical density

Various characteristics were significantly associated with seropositivity (>0.3 vs. ≤0.3 OD units), namely age (age 18–29 and ≥60 vs. 45–59 years), gender, and smoking status (Table 3). For increased likelihood of current/recent infection (≥1.0 vs. <1.0 OD units), only smoking status remained significant after backward elimination; although there was a trend towards increased likelihood of current/recent infection among those aged ≥60 years (Table 4).

**Table 3:** Estimated coefficients of the fitted logistic regression model for seropositivity,^a^ before and after backward elimination

Characteristics	Saturated model		Final model	
	OR (95% CI)	*p*-value	OR (95% CI)	*p*-value
Age 18–29 vs. 45–59 years^b^	1.64 (1.10–2.44)	.015	1.67 (1.13–2.46)	.0094
Age 30–44 vs. 45–59 years^b^	1.15 (0.78–1.70)	.48	1.17 (0.80–1.71)	.41
Age ≥60 vs. 45–59 years^b^	2.13 (1.46–3.12)	<.0001	1.97 (1.39–2.80)	.0002
Male vs. female	1.29 (1.00–1.67)	.050	1.30 (1.01–1.68)	.041
Vaccination yes vs. no	1.46 (0.92–2.29)	.11	–	–
Vaccination unknown vs. no	1.36 (0.90–2.05)	.15	–	–
Pertussis yes vs. no	0.99 (0.54–1.81)	.97	–	–
Pertussis unknown vs. no	0.99 (0.72–1.37)	.96	–	–
Medication^c^ yes vs. no	1.04 (0.71–1.54)	.83	–	–
Medication^c^ unknown vs. no	1.44 (0.62–3.33)	.39	–	–
Hospitalization^d^ yes vs. no	3.01 (0.79–11.55)	.11	–	–
Hospitalization^d^ unknown vs. no	1.03 (0.11–10.21)	.98	–	–
Current vs. never smoker	1.36 (0.99–1.87)	.055	1.38 (1.01–1.89)	.045
Former vs. never smoker	1.44 (1.07–1.95)	.018	1.46 (1.08–1.97)	.014

*Abbreviations: CI* confidence interval, *OD* optical density, *OR* odds ratio, *vs.* versus

^a^>0.3 OD units

^b^The age group with the lowest seropositivity was used as the reference

^c^Any antibiotics and/or other medication (i.e., any cough medicines) for lower respiratory tract infections or (suspected) pertussis infections in the previous 12 months

^d^Hospitalized due to respiratory infections in the previous 12 months

**Table 4:** Estimated coefficients of the fitted logistic regression model for increased likelihood of current/recent infection,^a^ before and after backward elimination

Characteristics	Saturated model		Final model	
	OR (95% CI)	*p*-value	OR (95% CI)	*p*-value
Age 18–29 vs. 45–59 years^b^	0.57 (0.10–3.31)	.53	0.58 (0.11–3.24)	.54
Age 30–44 vs. 45–59 years^b^	1.21 (0.31–4.74)	.79	1.21 (0.32–4.57)	.78
Age ≥60 vs. 45–59 years^b^	3.60 (1.06–12.24)	.040	2.99 (0.94–9.56)	.064
Male vs. female	1.55 (0.65–3.70)	.32	–	–
Vaccination yes vs. no	2.03 (0.35–11.81)	.43	–	–
Vaccination unknown vs. no	3.31 (0.71–15.5)	.13	–	–
Pertussis yes vs. no	0.45 (0.06–3.64)	.45	–	–
Pertussis unknown vs. no	0.30 (0.08–1.08)	.065	–	–
Medication^c^ yes vs. no	1.24 (0.35–4.38)	.74	–	–
Medication^c^ unknown vs. no	2.14 (0.25–17.97)	.49	–	–
Hospitalization^d^ yes vs. no	0.00 (0.00–NR)	.99	–	–
Hospitalization^d^ unknown vs. no	0.00 (0.00–NR)	1.00	–	–
Current vs. never smoker	7.08 (2.17–23.16)	.0012	7.50 (2.32–24.31)	.0008
Former vs. never smoker	4.12 (1.21–14.01)	.024	4.07 (1.21–13.64)	.023

*Abbreviations: CI* confidence interval, *NR* not reported, *OD* optical density, *OR* odds ratio, *vs.* versus

^a^≥1.0 OD units

^b^The age group with the lowest seropositivity was used as the reference

^c^Any antibiotics and/or other medication (i.e., any cough medicines) for lower respiratory tract infections or (suspected) pertussis infections in the previous 12 months

^d^Hospitalized due to respiratory infections in the previous 12 months

## Discussion

In this Hungarian study, 14.8% of the healthy adults tested (during 2014–2015) were seropositive (>0.3 OD units) for anti-PT IgG. This is lower than has been reported in seroprevalence studies in adults in other countries (approximately 20–80% [20–27]). However, it should be noted that these studies used a variety of different assays; and the units and cut-offs used to define seropositivity varied widely, which limits their comparability [16]. Further, seropositivity for anti-PT IgG can be due to prior pertussis infection or vaccination [28]. Although none of the subjects in the current study had been vaccinated against pertussis within the last year, this was not necessarily the case for the other seroepidemiological studies. Regardless of these issues, in the current study, 85.2% of the adults tested were seronegative and therefore at risk of pertussis infection. Although childhood vaccination rates in Hungary are very high [6], immunity to pertussis wanes over time [7–10], meaning that many adults are no longer protected. This could be overcome by the introduction of adult pertussis boosters, an approach that has already been adopted in various European countries [11] and the US [29].

In the current study, 1.1% of subjects had anti-PT IgG levels ≥1.0 OD (indicative of current/recent infection). It is difficult to compare this with other studies, which have used a range of different assays, cut-offs, and definitions, but infection within the last year/recent/active infection rates of approximately 1–10% have been reported in adults [22, 25, 27, 30–36], which are similar to or higher than in the current study.

In order to try to compare our seroprevalence results with notified cases of pertussis, an estimation of pertussis incidence based on seroprevalence data combined with the manufacturer pre-defined cut-off values was undertaken. Previously, de Melker et al. [37] used seroprevalence data combined with antibody decline over time after infection to estimate incidence. Assuming that “recent” relates to the previous year, 2/1999 subjects (0.1%) with a level strongly indicative of current/recent infection (≥1.5 OD units) in the current study would translate into a crude annual incidence of pertussis infection of 100/100,000. Similarly, the 22/1999 subjects (1.1%) with a level indicative of current/recent infection (≥1.0 OD units) would give a crude annual incidence of 1100/100,000. These crude incidences are roughly 500- and 6000-fold higher than the notified rate among those aged ≥20 years in Hungary in 2014 (approximately 0.19/100,000) [13], indicating a very high level of underreporting. A recent review has found similar discrepancies all around the world [16]. However, it should be noted that the current study was not designed to perform this type of calculation and “recent” (as defined by the manufacturer) may not equate to 1 year. Therefore, these estimations and comparisons should be interpreted with caution.

The introduction of adult pertussis boosters could help alleviate the burden of pertussis in this age group. It may also help reduce the risk of transmission to susceptible infants. Other approaches to protect infants prior to their primary immunizations include cocooning or immunization of pregnant women. Various European countries [11] and the US [29] have introduced one or other of these approaches to help protect newborns. However, recent data indicate that post-partum vaccination of parents may not be as successful as maternal vaccination during pregnancy [38–40]. Also, although maternal immunization may benefit newborns, this approach alone would not address the burden of pertussis in the adult population.

In the current study, seropositivity was significantly more likely among those aged ≥60 and 18–29 years vs. 45–59 years; and there was a trend towards more likely current/recent infection among those aged ≥60 years vs. 45–59 years. Overall, these results support waning immunity following childhood vaccinations, ultimately resulting in higher levels of infection during later years. The pertussis booster at age 11 years was introduced in Hungary in 2006 [4], so some of the adults in the youngest age group could have received this vaccination approximately 7 or 8 years earlier. The remainder of the population would probably have last received pertussis vaccination ≥14 years ago; or never have been vaccinated. Although immunity wanes quite quickly after pertussis vaccination [41], anti-PT antibodies can persist for up to approximately 10 years [42], therefore the elevated rate of seropositivity in the 18–29-year age group is likely due to adolescent (or even childhood) vaccination. Among those aged ≥60 years, the elevated seropositivity is likely related to pertussis infection, which was also elevated in this age group. Based on these results, vaccination of the elderly could be beneficial in Hungary to address the pertussis burden in this age group, who can experience considerable pertussis-related morbidity [43].

Some other studies have also reported higher seropositivity among older adults [20, 23, 27]. However, others have reported highest seropositivity rates in various younger adult age groups [21, 24] or little variation by age among adults [25]. Similarly, some previous studies have reported a possible link between likely pertussis infection and advancing age [20, 22, 31], but others have reported no such link [25, 34, 35]. Whether these results are indicative of true differences between countries and/or years or whether they are due to a lack of sufficient power to detect such differences is unclear.

Regarding gender, we found that males were significantly more likely to be seropositive than females; but not significantly more likely to have anti-PT IgG levels indicative of current/recent infection. Higher male seropositivity has also been reported in studies from Mexico (*p* = .0007) [24] and Korea (*p* = .023) [23]; but studies from Spain [26], Greece [21], and The Gambia [22] have reported no significant differences in seropositivity between genders. Regarding higher male likelihood of recent infection, this has been reported to be significant in a study from The Netherlands [31], non-significant in a study from Denmark [34], higher but significance not reported in one from The Gambia [22], or no significant gender differences in two studies from China [35, 36]. Overall, it is unclear whether there is a true link between pertussis and gender and, if there is, the cause is unknown.

Regarding smoking status, we found correlations between seropositivity and current/former smoking, and even stronger correlations between evidence of recent pertussis infection and current/former smoking. This could be because the epithelial cells of smokers have been shown to have enhanced binding to *Bordetella pertussis* [44], thus increasing pertussis infection risk. However, a seroepidemiology study in Denmark found no significant link between smoking status and recent pertussis infection [34].

## Limitations

Some of the subjects were enrolled into this study at a regular GP visit, so the studied population may be sicker and/or more health-conscious than the general population. This method of recruitment probably also accounts for the higher proportion of female subjects. Unfortunately, data on the route of recruitment (GP visit, telephone call or email) were not collected, so it is not possible to compare results by route of recruitment. Also, although we recognize that this “convenience sample” is a limitation of this study, we feel that it is likely representative of the general Hungarian adult population.

As there is no agreed threshold of anti-PT antibodies that provide protection, seroprevalence studies are notoriously difficult to interpret. Also, the manufacturer’s definition of “current/recent” infection is rather vague, making comparisons with other studies difficult. Lastly, *p-*values were not adjusted for multiplicity of endpoints (as there was only one endpoint of interest), so the statistical significance of the results should be interpreted with caution.

## Conclusions

In this seroprevalence study, 14.8% of adults in Hungary were found to be seropositive for anti-PT IgG antibodies, with increased risk among males, those aged ≥60 or 18–29 years, and current/former smokers. The remaining 85.2% of adults were seronegative, and therefore at risk of pertussis infection. Furthermore, 1.1% of adults had evidence of current/recent pertussis infection, with increased risk among current/former smokers and a trend towards an increased risk among those aged ≥60 years. This gives an estimated crude annual pertussis incidence of 1100/100,000, roughly 6000-fold higher than the notified rate in Hungary. This indicates that many adults in Hungary may have undiagnosed pertussis, and this could be unknowingly transmitted to susceptible infants. The introduction of additional pertussis boosters during adulthood could help overcome this potential issue.

## Abbreviations

Anti-PT IgG: Anti-pertussis toxin immunoglobulin G
CI: Confidence interval
ELISA: Enzyme-linked immunosorbent assay
EU: ELISA units
GP: General practitioner
OD: Optical density
SD: Standard deviation
US: United States
